# FGFR3 promotes the growth and malignancy of melanoma by influencing EMT and the phosphorylation of ERK, AKT, and EGFR

**DOI:** 10.1186/s12885-019-6161-8

**Published:** 2019-10-16

**Authors:** Lei Li, Shuai Zhang, Hao Li, Haiyan Chou

**Affiliations:** 1 Department of Plastic and Cosmetic Surgery, Henan Provincial People’s Hospital, People’s Hospital of Zhengzhou University, Zhengzhou, 450003 Henan China; 2 Department of Breast Surgery, The First Affiliated Hospital of Zhengzhou University, Zhengzhou, Henan China

**Keywords:** FGFR3, Melanoma, Metastasis, Epithelial-mesenchymal transition, ERK, AKT, EGFR

## Abstract

**Background:**

Overexpression of fibroblast growth factor receptor 3 (FGFR3) has been linked to tumor progression in many types of cancer. The role of FGFR3 in melanoma remains unclear. In this study, we aimed to uncover the role of FGFR3 in the growth and metastasis of melanoma.

**Methods:**

FGFR3 knockdown and overexpression strategies were employed to investigate the effects of FGFR3 on colony formation, cell apoptosis, proliferation, migration, and in vitro invasion, along with the growth and metastasis of melanoma in a xenografts mouse model. The protein expression levels of extracellular signal-regulated kinase (ERK), protein kinase B (AKT), epidermal growth factor receptor (EGFR), and epithelial-mesenchymal transition (EMT) markers were determined by Western blot analysis.

**Results:**

The mRNA expression of FGFR3 was higher in melanoma tissues than normal healthy tissues. FGFR3 expression in cutaneous malignant melanoma (CMM) tissues was positively correlated with the Breslow thickness and lymph node metastasis. In A357 cells, knockdown of the *FGFR3* gene decreased the colony formation ability, cell proliferation, invasion, and migration, but increased the caspase 3 activity and the apoptosis rate; overexpression of FGFR3 increased the colony formation ability, cell proliferation, invasion, and migration, but decreased the caspase 3 activity and apoptosis rates. FGFR3 knockdown also upregulated E-cadherin, downregulated N-cadherin and vimentin, and decreased the phosphorylation levels of ERK, AKT, and EGFR. In the MCC xenografts mice, knockdown of FGFR3 decreased tumor growth and metastasis.

**Conclusions:**

FGFR3, which is highly expressed in CMM tissues, is correlated with increased Breslow thickness and lymph node metastasis. FGFR3 promotes melanoma growth, metastasis, and EMT behaviors, likely by affecting the phosphorylation levels of ERK, AKT, and EGFR.

## Background

Cutaneous malignant melanoma (CMM), which comprises 65% of all skin cancers, is a highly lethal form of skin cancer. CMM ranks as the sixth most common cancer in both males and females in the United States, and it occurs more frequently in patients with lighter skin than those with darker skin. Several factors contribute to the formation of melanoma, including exposure to ultraviolet (UV) radiation and the malignant transformation of moles, along with a variety of genetic factors. Early detection and surgical intervention, in combination with immunotherapies, radiation therapy, and chemotherapy, are essential to successfully treat and prevent the spread of CMM to other vital organs and tissues, such as the brain, liver, lungs, and bones. The malignancy is more likely to spread in patients with deep primary tumors or regional lymph node metastases, which leads to a median survival of only 6–9 months, and a dismal 5-year survival rate of less than 5% [1].

Significant progress has been made in the identification of genetic markers and cellular pathways involved with the development of melanoma and the potential mechanisms by which melanoma acquires resistance to the current therapies [2]. However, melanoma remains a lethal disease, and new diagnostic and treatment options are needed to improve patient outcomes in the clinic.

The fibroblast growth factor receptors (FGFR) comprise a family of transmembrane tyrosine kinase receptors [3] that play vital roles in cell differentiation, growth, and angiogenesis through binding of their respective ligands [4, 5]. Activation of FGFR results from dimerization of the receptor monomers and transphosphorylation of the kinase domain loop tyrosine residues. Activation of FGFR modulates the cytoplasmic downstream molecules and contributes to its carcinogenesis through signal transducer and activator of transcription protein (STAT), phosphatidylinositide 3-kinases/protein kinase B (PI3K/AKT), and RAS/mitogen activated protein kinase (RAS/MAPK) pathways [4, 6].

Overexpression of FGFR3 has been associated with several types of cancer, including multiple myeloma, bladder cancer, non-small cell lung cancer, oral cancers, and oropharyngeal squamous cell carcinoma [7–10]. An FGFR3 activation mutation increased the invasiveness of many tumors, making it a potential target for therapeutic intervention. All four subtypes of FGFR, including FGFR1, FGFR2, FGFR3, and FGFR4 are involved in the genesis of neoplastic skin lesions. Amplification of the *FGFR1* gene and its overexpression in squamous cell carcinomas (SCC) has been shown to augment keratinocyte proliferation and tumor progression [11]. In addition, FGFR1 plays a key role in the growth, angiogenesis, distant migration, and metastasis of melanomas [12, 13]. FGFR2 was unchanged in SCC. However, keratinocyte-specific deletion of the *FGFR2* gene made mice more sensitive to chemical carcinogenesis, suggesting that FGFR2 may function as a tumor suppressor [14]. Also, FGFR2 promotes the metastasis of melanoma cells via store-operated calcium entry [15]. FGFR3 activation mutations have been connected to keratosis and epidermal nevus in patients [16]. The FGFR3-TACC3 (transforming acidic coiled-coil containing protein 3) fusion protein has been detected in patients with malignant melanoma [17]. In addition, some FGFR3 mutations have been associated with an improved prognosis and decreased risk of metastasis in epithelial tumors, including bladder carcinomas [18–20]. However, the same FGFR3 activation mutations have been associated with disease progression in some hematopoietic malignancies [21, 22]. In addition to FGFR3, FGFR4 expression has been correlated with the metastasis of melanoma in patients [23].

Both FGFR and EGFR modulate the PI3K/Akt and ERK signaling pathways [4, 24, 25]. Activation of the PI3K/Akt and ERK signaling pathways promotes the growth [4, 24, 25] and epithelial-mesenchymal transition (EMT) in many aggressive forms of cancer [26]. However, the role of FGFR3 in melanoma has not been elucidated. In this study, we investigated the role of FGFR3 in the growth and metastasis of melanoma using FGFR3 knockdown and overexpression strategies in vitro and in vivo.

## Methods

### Materials

The primary anti-FGFR3 antibody was purchased from Abcam (Cambridge, United Kingdom). The anti-E-cadherin, anti-N-cadherin, anti-vimentin, anti-ERK, anti-AKT, anti-EGF, anti-phosphorylated ERK, anti-phosphorylated AKT, and anti-phosphorylated EGF antibodies were obtained from Cell Signaling Technology (Danvers, MA, USA). The HRP-conjugated sheep anti-rabbit and sheep anti-mouse secondary antibodies were obtained from Thermo Fisher Scientific (Waltham, MA, USA).

### Patients and tissue collection

All procedures in this study were approved by the Henan Provincial People’s Hospital Ethics Committee. Forty-two patients with CMM who had received free treatment in the Department of Plastic and Cosmetic Surgery at the Henan Provincial People’s Hospital (China) from 2016 to 2018 were recruited for this study. All patients were required to provide written informed consent. Patients were excluded for any of the following criteria: (1) tumor present in multiple sites or organs; (2) actively being treated with radiation therapy or chemotherapy; and (3) patient refused to participate. The demographic characteristics of the participants are shown in Table 1. Tumor and healthy tissue were cut into small pieces and placed into separate cryogenic storage tubes for storage at − 80 °C for future experiments. For gene expression studies, some tissue pieces were placed in a solution of RNAlater (Thermo Fisher Scientific). Tissues for histology and immunohistochemistry (IHC) studies were fixed in formalin.

**Table 1:** Relationship of FGFR3 with different clinicopathologic parameters of melanoma patients

Parameters	FGFR3 expression			
	n	Low	High	*P*
Age				
≤ 60	29	19	10	NS
> 60	13	7	6	
Gender				
Male	25	17	8	NS
Female	17	9	8	
Location				
Head/neck	2	1	1	NS
Trunk	29	18	11	
Extremities	8	5	3	
Feet	3	2	1	
Breslow thickness				
1.01–2.00	14	12	2	0.0119
2.01–4.00	15	6	9	
> 4.00	13	8	5	
Clark Classification				
I-II	9	6	3	NS
III-IV	33	20	13	
Ulceration				
Yes	20	10	10	NS
No	22	16	6	
Nodular				
Yes	17	9	8	NS
No	25	17	8	
SLN status				
Not involved	27	21	6	0.0045
Involved	15	5	10	

Note: *NS* No significance, *SNL* Sentinel lymph node

### Hematoxylin and eosin (H&E) staining and immunohistochemistry

H&E staining was performed according to previously described procedures [27]. Briefly, the tissues were removed from formalin and dehydrated using a series of increasing ethanol concentrations. Next, the tissue blocks were cleared in xylene and embedded into paraffin blocks. Paraffin sections of 4 μm were made and the paraffin was removed with xylene. Then, the sections were hydrated in a descending gradient of alcohol solutions from 100 to 75%, followed by water. Sections were stained with hematoxylin for 5 min, rinsed in water and differentiated in hydrochloric acid in alcohol for 30 s. After rinsing in water for 3 min, the sections were stained with eosin for 2 min. The sections were dehydrated in an upgrading gradient of alcohol (75–100%), cleared in a mixture of xylene/phenol (3:1) for 1 min, and followed by xylene for 1 min. All of the slides were mounted using neutral resin.

### Immunohistochemistry

Paraffin sections of 4 μm were dewaxed, hydrated in solutions of descending ethanol concentration (100–75%), and rinsed with water. Slides were boiled in a sodium citrate solution for 20 min. After cooling down to room temperature, they were rinsed in phosphate buffer saline (PBS) that contained three drops of 0.3% H_2_O_2_ (Sangon, Shanghai, China) and incubated in the dark for 10 min. After being rinsed in PBS, the slides were blocked with goat or rabbit serum for 20 min. After the serum was removed, 50 μl of the primary antibody was added to the slide, and the slides were incubated at 4 °C overnight. The slides were washed with PBS twice for 5 min each before the secondary antibody was added, and the slides were incubated at room temperature for 30 min. After the incubation, the slides were washed in PBS twice for 5 min each time before the SAB solution was added and incubated in the dark for 30 min. After being washed and DAB was added, the slides were incubated at room temperature and examined under a microscope to determine when to stop the reaction. Next, the slides were stained with hematoxylin for 30 s and rinsed in tap water for 15 min. Before mounting, the sections were dehydrated using an increasing gradient of alcohol (70–100%), which was followed by xylene and neutral resin for mounting.

FGFR3 expression was quantified by calculating the staining intensity and the number of positively stained cells. Scores were assessed for the intensity of staining with zero designated as negative, one designated as weak staining, two designated as moderate staining, and three designated as strong staining. Scores were also accessed on the number of positively stained cells with zero indicative of no cells, one designated as 1–25% cells positively stained, two designated as 26–50% cells positively stained, and three designated as 51–100% cells positively stained. FGFR3 expression was represented by the result derived from multiplying the two scores from the same slide. Scores of 0–3 were taken as low expression, while scores of 4–9 were taken as high expression.

### Cell culture

The malignant melanoma cell line A375 was obtained from the American Type Culture Collection (cat. No.: CRL-1619, ATCC, Manassas, VA, USA) and were cultured according to the supplier’s protocol in a humified incubator with a constant temperature.

### Real-time RT-PCR

RNA was extracted from tissues and cells with Trizol (Thermo Fisher Scientific) according to the manufacturer’s protocol. Briefly, the tissues were frozen in liquid nitrogen before being ground to powder. The powder was transferred to an Eppendorf tube containing 1 ml of Trizol. Next, 200 μl of chloroform was added to the tube. The tube was mixed thoroughly and stored at room temperature for 10 min. Next, the tube was vortexed for 15 s and centrifuged at 12,000 rpm for 20 min. The upper aqueous phase was transferred to another Eppendorf tube that contained 500 μl of isopropanol. The tube was mixed and allowed to remain at room temperature for 10 min. After centrifugation at 12,000 rpm for 10 min, the supernatant was discarded, and the pellet was washed twice with 1 ml of 75% ethanol. Again, the tube was centrifuged at 12,000 rpm for 5 min and the supernatant was discarded. The pellet was air-dried and dissolved in diethyl pyrocarbonate (DEPC)-treated ddH_2_O. The concentration of RNA was measured with a spectrophotometer. For the cells adhered to the wells of the plate, 1 ml of trizol was added to the tube, which was mixed slowly for 5 min to dissolve the cells. The homogenate was transferred to an Eppendorf tube, and the RNA was extracted using the same procedures.

cDNA was synthesized using reverse transcription. The RNA solution of 100 ng/μl was incubated at 65 °C for 5 min and immediately placed on ice. The reverse transcription reaction was performed in a 10 μl reaction mixture containing 6 μl of nuclease-free water, 2 μl of 5 × RT Buffer, 0.5 μl of RT Enzyme Mix, 0.5 μl of Primer Mix, and 1 μl of RNA at 37 °C. After 15 min, the reaction was terminated by incubation at 98 °C for 5 min. The synthesized cDNA was stored at − 20 °C.

FGFR3 mRNA was quantified by real-time PCR using the SYBR® Green Real-time PCR Master Mix (TOYOBO, Japan) according to the manufacturer’s instruction. The sequences of the primers used are shown in Table 2. The PCR reaction occurred in 50 μl of reaction mixture containing 16 μl of ddH_2_O, 25 μl of SYBR® Green, 2 μl of the forward primer, 2 μl of the backward primer, and 5 μl of cDNA. The PCR profile was as follows: 1 cycle, 95 °C for 60 s; 40 cycles, 95 °C for 15 s; and 60 °C for 60 s. GAPDH was used as the internal standard. The relative expression level of FGFR3 was represented by 2^-ΔΔCT^*.*

**Table 2:** Sequences of the primers used for RT-PCR

Name	Label	Sequence
FGFR3	Forward	5′-TGCGTCGTGGAGAACAAGTTT-3’
	Reverse	5′-GCACGGTAACGTAGGGTGTG-3’
GAPDH	Forward	5′-AGCCACATCGCTCAGACAC-3’
	Reverse	5′-GCCCAATACGACCAAATCC-3’

### Establishment of a stable transgenic cell line

A375 cells were seeded into the wells of a six-well plate. Once the cells reached 60% confluence, they were transfected with the chronic virus. The media was changed after 24 h, and the transfected cells were treated with puromycin. The stable transfected cells were selected when the fluorescence ratio was greater than 95%.

### Transient transfection

Cells at the exponential phase were treated with trypsin, suspended and seeded into a six-well plate. The media were removed once the cells grew to 65% confluence. After the wells were rinsed with PBS, 1.5 ml Gibco opti-MEM was added to each well and 500 μl of the transfection mixture, which contained 10 μl of Lipofectamine 2000 (Thermo Fisher Scientific), 5 μg pcDNA3.0-FGFR3 or the control plasmids, and 490 μl of Gibco opti-MEM, was added drop-wise to the wells. After incubation for 6 h, the media was changed. After incubation for another 48 h, FGFR3 expression was detected using an automated plate reader and the cells were used for functional tests.

### Cell proliferation assay

One-thousand cells were seeded into each well of a 96 well plate. At desired time points, 10 μl of CCK-8 (DOJINDO, China) was added to each well. After incubating for 2 h in the dark, the light absorption at 450 nm was measured using a microplate reader.

### Caspase 3 activity assay

A standard curve was made with pNA according to the manufacturer’s protocol. First, the cells were treated with trypsin and pelleted via centrifugation. The cell pellet was resuspended in 100 μl of lysis buffer and incubated in a water bath for 15 min. Next, the lysate was centrifuged at 16,000 g at 4 °C for 15 min, and the supernatant was transferred to a pre-cooled tube. The reaction tubes contained 100 μl of solution, which consisted of 40 μl of buffer, 50 μl of the protein sample, and 10 μl of Ac-DEVD-pNA (2 mM). The samples were incubated for 2 h at 37 °C. For the control, the same reaction was performed using the same conditions, but replacing the 50 μl of protein sample with 50 μl of lysis buffer. The absorption was measured at 405 nm using a microplate reader, and caspase-3 activity was calculated using the pNA standard curve. After the protein concentration was tested using the Bradford assay, the caspase-3 activity was converted into caspase-3 activity units/unit of protein.

### Colony formation test

A375 cells at exponential phase of growth were suspended in media as a single cell suspension at a density of 500 cells/ml. Next, 2 ml of the cell suspension was added to each well of a six-well plate and incubated at 37 °C in an incubator containing 5% CO_2_ for 15 days_._ After the media were discarded, the cells were stained with 1% crystal violet in methyl for 30 min. Photos were taken after the cells being washed with tap water to determine the number of cell colonies.

### Cell apoptosis assay

After washing the cells twice with PBS via centrifugation at 1000 rpm for 10 min, cell apoptosis was detected using the Annexin V-FITC/PI Apoptosis Detection Kit (BD Bioscience, San Jose, CA, USA) according to the manufacturer’s instructions. Both AV-FITC and PI were added to the cell pellets. No AV-FITC or PI was added to create the negative controls, while AV-FITC or PI was added to create the two positive controls. After being mixed, the samples were incubated at room temperature in the dark for 15 min. Next, 300 μl of 1x Binding Buffer was added to each tube, and cell apoptosis was measured by flow cytometry.

### Cell migration and invasion test

Cells were suspended in media without FBS at a density of 2.5 × 10^5^ cells/ml. After 200 μl of cell suspension was added to the upper chamber of a transwell plate, 600 μl of medium containing FBS was added to the bottom chamber of the transwell plate. After being cultured for 48 h, the transwell chambers were removed, and the media in the upper chamber was discarded. The chambers were stained with 1% crystal violet for 30 min and rinsed gently. After the cells adhered to the wells were removed using 200 μl Eppendorf tips, the migrated cells were visualized and counted under a standard microscope. Cell inversion was tested using Matrigel (Corning Inc., Corning, NY, USA) invasion chambers following the same procedures described above.

### Western blot analysis

Cells adhered to walls were washed with cold PBS twice. A mixture of protease and phosphatase inhibitors (ratio 100:1) was added to the plates to remove and lyse the cells on ice for 30 min. The lysate was transferred into a 1.5 ml Eppendorf tube and sonicated with ultrasound three times for 20 s each. For protein extraction from the tissues, xenografts from the LV-control group and LV-shFGFR3 group were harvested and flash frozen in liquid nitrogen. When ready, the frozen tissues were cut into 2 mm pieces and grounded using glass mortar and pestle in 1 mL of lysis buffer. The mixture was further homogenized, centrifuged, and the supernatant was collected. The proteins were pelleted by centrifugation at 12,000 rpm for 30 min at 4 °C. The protein concentration was determined using the Pierce BCA Protein Assay Kit (Thermo Fisher Scientific) and adjusted to the same for all samples. Proteins were separated by SDS-PAGE electrophoresis and transferred onto a PVDF membrane. The membrane was blocked in 5% milk, followed by incubation with the corresponding primary antibody and the secondary antibody. The signals were developed in ECL (Thermo Fisher Scientific), captured by autoradiography and quantified with a densitometer.

### Growth and metastasis of melanoma in vivo in mice xenografts

Procedures with animals were approved by the Animal Ethics Committee of Zhengzhou University. A total of 20 male BALB/c nude mice (age, 4 weeks old; weight, 16–20 g) were purchased from the Institute of Zoology, Chinese Academy of Science (Shanghai, China). The mice were maintained under specific pathogen-free (SPF) conditions according to the institutional guidelines for animal welfare. The stable FGFR3 knockdown A375 cells and control A375 cells were separately suspended at a density of 4 × 10^7^ cells/ml. Each mouse was injected with 0.1 ml of the cell suspension subcutaneously into the right flank. The mice were monitored for the presence and size of tumors weekly. Five weeks after inoculation, the animals were sacrificed by cervical dislocation, and the tumors were excised for analysis. For testing the metastasis of cancer cells to the lungs of the nude mice, 5 × 10^6^ cells in 0.1 ml of cell suspension were injected into each mouse through the tail vein. The mice were sacrificed by cervical dislocation 2 months after the injection for extraction of the lung tissues. After the number of tumors was determined, the lung tissues were fixed in formalin for H&E staining.

### Statistical analysis

Statistical analysis was performed using the SPSS software package (IBM, Chicago, IL, USA). The statistical difference between two groups was examined by the Student’s *t*-test. Multiple comparisons were accessed using the one-way analysis of variance (ANOVA). All tests were performed in triplicate, and the values were expressed as the mean ± standard deviation (SD). *P*-values < 0.05 were considered as statistically significant.

## Results

### Expression of FGFR3 was high in melanoma tissues and altered by the gene intervention strategies in A357 cells

**Fig. 1:**
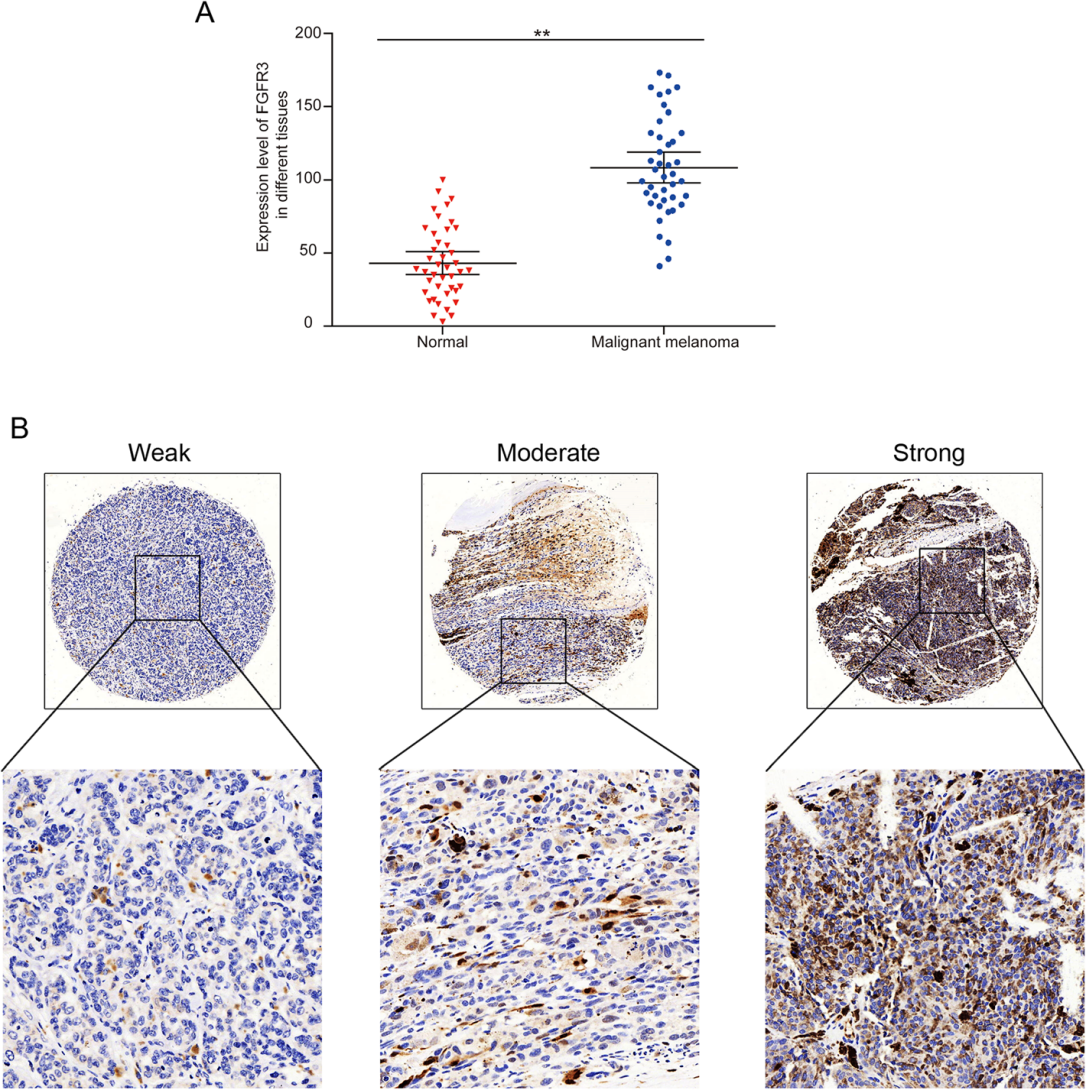
Expression of FGFR3 in melanoma tissues. **a** the mRNA level of FGFR3 in malignant melanoma tissues and corresponding adjacent normal tissues was evaluated by qRT-PCR (*n* = 4, **p < 0.01); **b** FGFR3 protein expression in melanoma tissues obtained from 42 patients was detected by immunohistochemical staining. The FGFR3 staining was categorized as weak, moderate, or strong (upper row, 40x), which were further magnified (lower row, 200x). The FGFR3 expression levels were used to evaluate the relationship between FGFR3 expression and the clinicopathological parameters of the malignant melanoma patients

The mRNA expression level of FGFR3 was significantly higher in melanoma tissues than the surrounding normal healthy tissues (Fig. 1a). The protein expression of FGFR3 in the malignant melanoma tissues was correlated with the Breslow thickness and lymph node metastasis (*p* < 0.05) (Fig. 1b).

**Fig. 2:**
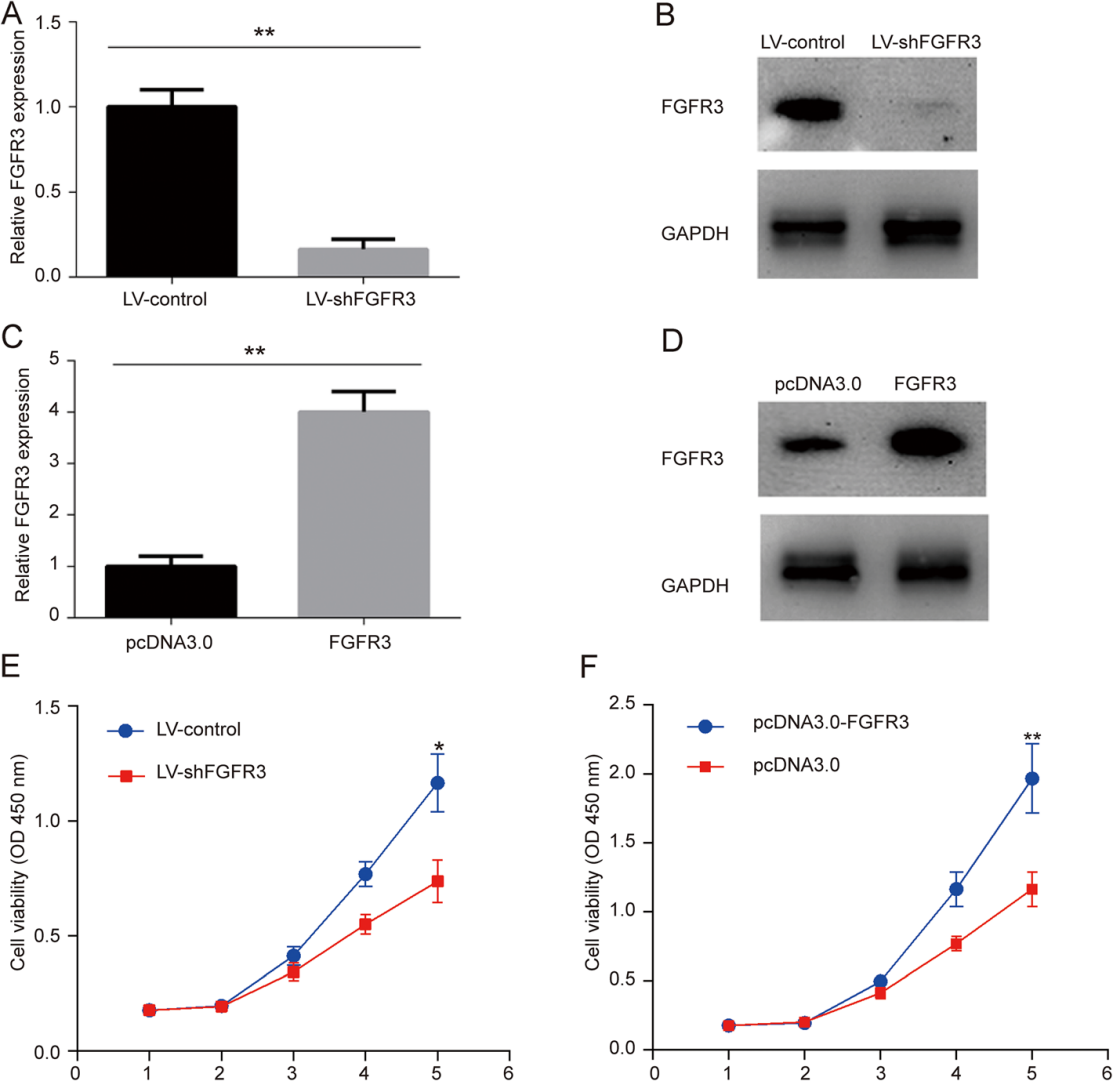
Expression of FGFR3 and the proliferation ability in A357 cells transfected with a lentivirus containing shFGFR3 or pcDNA3.0-FGFR3. **a** comparison of GFR3 mRNA levels in A357 cells transfected with lentivirus containing shFGFR3 with A357 cells transfected with a control vector; **b** GFR3 protein was detected by Western blot in A357 cells transfected with lentivirus containing shFGFR3 and A357 cells transfected with a control vector; **c** comparison of FGFR3 mRNA levels in A357 cells transfected with pcDNA3.0-FGFR3 with A357 cells transfected with a control plasmid; **d** GFR3 protein detected by Western blot in A357 cells transfected with pcDNA3.0-FGFR3 and A357 cells transfected with control plasmids; **e** time course of the viability of A357 cells transfected with LV-shFGFR3 or LV-control; **f** time course of the viability of A357 cells transfected with pcDNA3.0-FGFR3 or the control plasmid (* *p* < 0.05; * * *p* < 0.01)

Transfection of A357 cells with LV-shFGFR3 significantly (*p* < 0.05) decreased the mRNA (Fig. 2a) and protein (Fig. 2b) expression levels of FGFR3 as compared with the non-transfected controls. Transient transfection of A357 cells with pcDNA3.0-FGFR3 significantly (*p* < 0.05) increased the mRNA (Fig. 2c) and protein (Fig. 2d) expression levels of FGFR3 as compared with the cells transfected with the control plasmid.

### FGFR3 promoted the growth, colony formation, migration, and invasion abilities of melanoma A357 cells in vitro

**Fig. 3:**
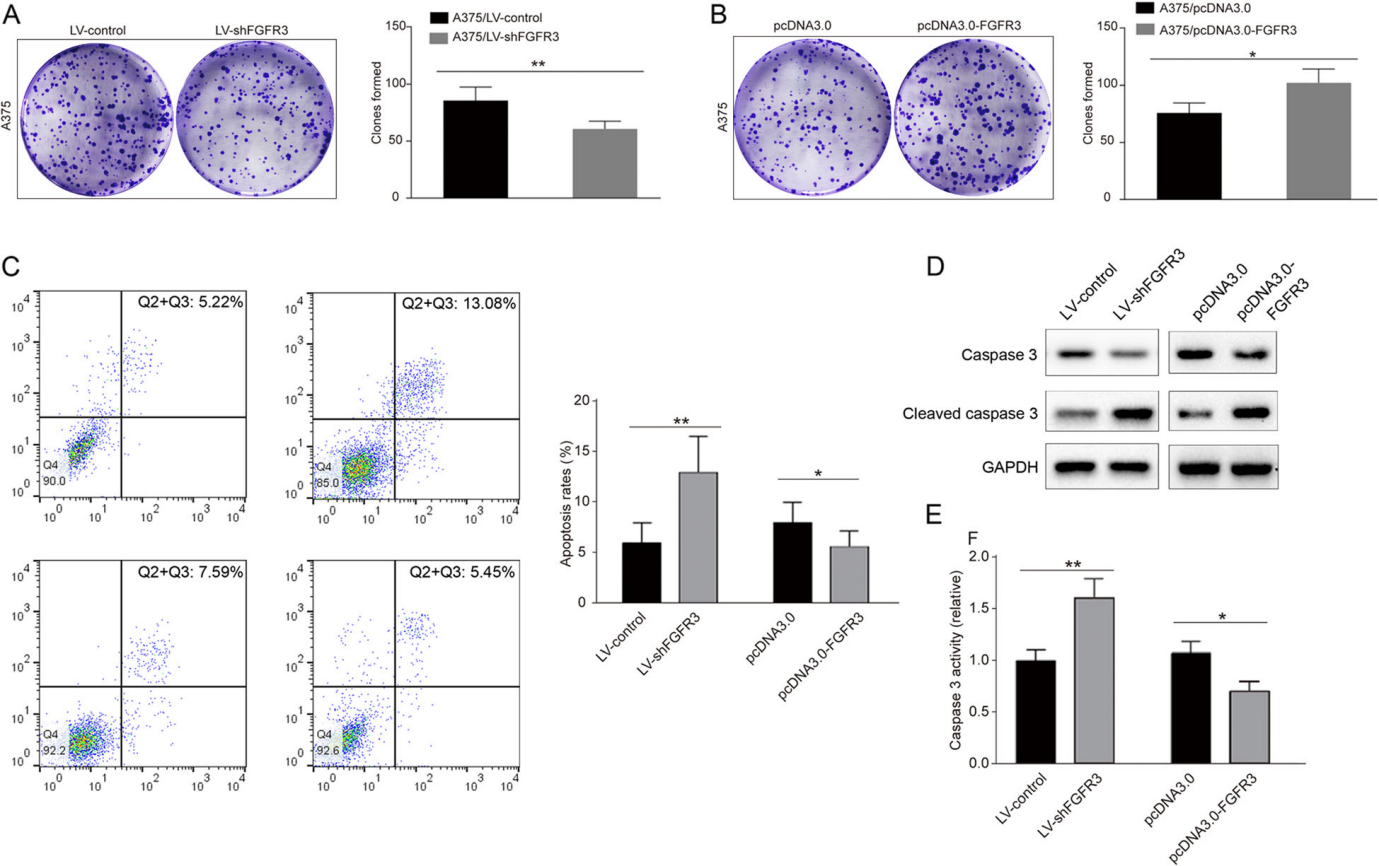
The colony formation ability and apoptosis rate of A357 cells. **a** left panel, colonies formed by A357 cells transfected with LV-shFGFR3 or LV-control; right panel, comparison of the numbers of colonies formed by A357 cells transfected with LV-shFGFR3 or LV-control; **b** left panel, colonies formed by A357 cells transfected with pcDNA3.0-FGFR3 or the control plasmids; right panel, comparison of the numbers of colonies formed by A357 cells transfected with pcDNA3.0-FGFR3 or the control plasmids; **c** left panel, dot-plots of cell apoptosis measured by flow cytometry; right panel, comparison of the apoptosis rates among A357 cells transfected with LV-shFGFR3, LV-control, pcDNA3.0-FGFR3, or the control plasmids for pcDNA3.0-FGFR3; **d** Western blot analysis of different forms of caspase3; **e** comparison of caspase 3 activity in A357 cells transfected with LV-shFGFR3, LV-control, pcDNA3.0-FGFR3, or the control plasmids (* *p* < 0.05;* * *p* < 0.01)

Knockdown of FGFR3 significantly (*p* < 0.05) inhibited the proliferation of the A357 cells in vitro (Fig. 2e), while FGFR3 overexpression significantly (*p* < 0.05) increased the proliferation of these cells (Fig. 2f). Knockdown of FGFR3 significantly (*p* < 0.05) increased the apoptosis rate, while FGFR3 overexpression significantly decreased the apoptosis rate of melanoma cells in vitro (Fig. 3e-f). Knockdown of FGFR3 significantly (*p* < 0.05) increased the activity of caspase-3, while FGFR3 overexpression significantly (*p* < 0.05) decreased the activity of caspase-3 in A357 cells in vitro (Fig. 3g-h).

Knockdown of FGFR3 significantly (*p* < 0.05) decreased the number of colonies formed by the A357 cells transfected with LV-shFGFR3, as compared with the cells transfected with LV-control (Fig. 3a-b). Overexpression of FGFR3 significantly (*p* < 0.05) increased the number of colonies formed by the A357 cells transfected with pcDNA3.0-FGFR3, as compared with the cells transfected with pcDNA3.0 (Fig. 3c-d).

**Fig. 4:**
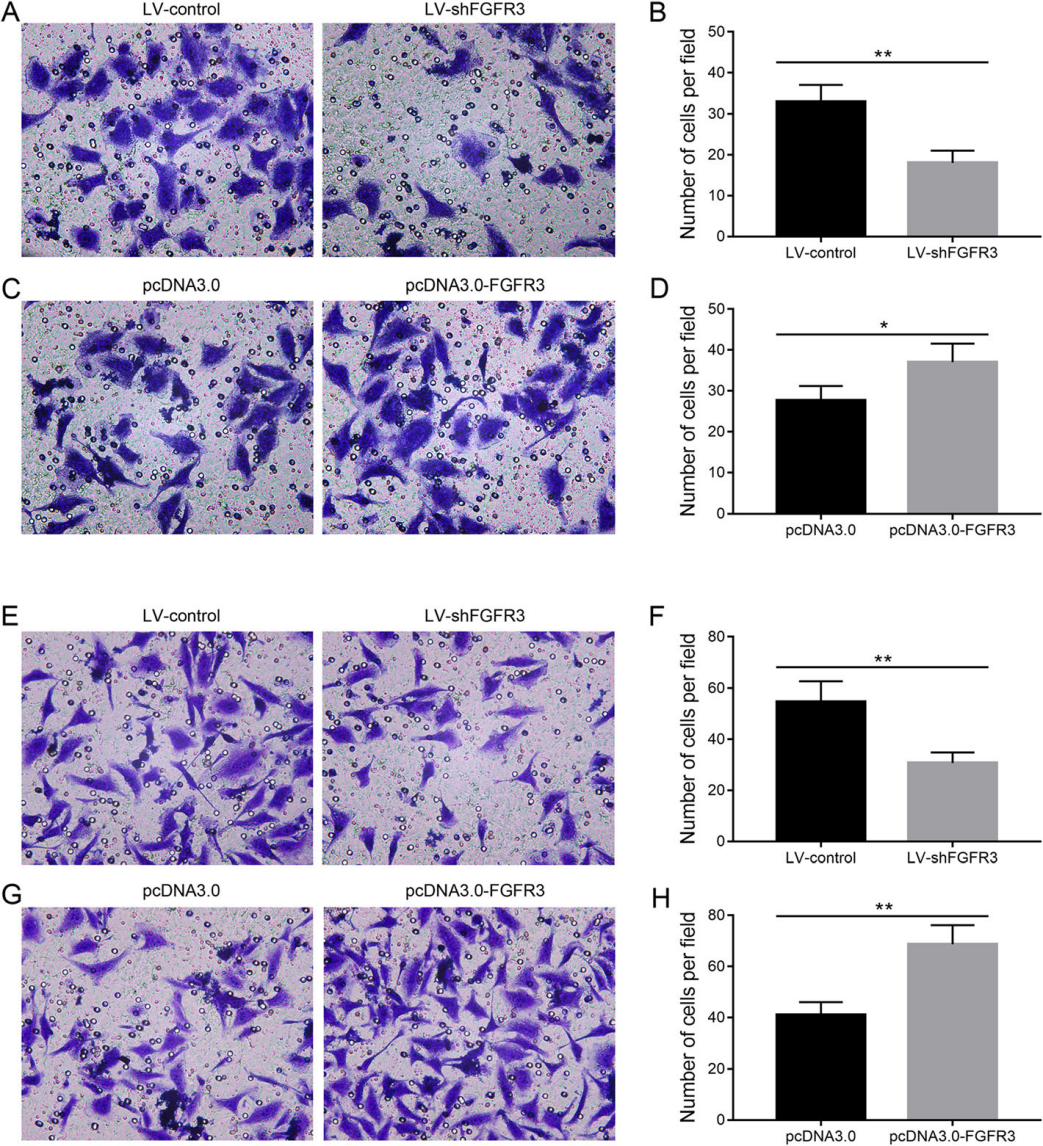
The migration and invasion abilities of A375 cells in vitro. **a** cell migration from the A357 cells transfected with LV-shFGFR3 or LV-control; **b** comparison of the numbers of migrating cells from the A357 cells transfected with LV-shFGFR3 or LV-control; **c** cells migration from the A357 transfected with pcDNA3.0-FGFR3 or the control plasmids; **d** comparison of the numbers of cells that migrated from the A357 cells transfected with pcDNA3.0-FGFR3 or the control plasmids; **e** cells invaded from the A357 cells transfected with LV-shFGFR3 or LV-control; **f** comparison of the numbers of invading cells from the A357 cells transfected with LV-shFGFR3 or LV-control; **g** cells invaded from the A357 transfected with pcDNA3.0-FGFR3 or the control plasmids; **h** comparison of the numbers of cells that invaded from the A357 cells transfected with pcDNA3.0-FGFR3 or the control plasmids (* *p* < 0.05; ** *p* < 0.05)

Knockdown of FGFR3 significantly (*p* < 0.05) reduced the number of cells that migrated (Fig. 4a-b) and the number of cells that invaded (Fig. 4e-f) in vitro, while FGFR3 overexpression significantly (*p* < 0.05) increased the number of cells that migrated (Fig. 4c-d) and the number of cells that invaded (Fig. 4g-h).

### FGFR3 knockdown reduced in vivo melanoma growth and metastasis to lung

**Fig. 5:**
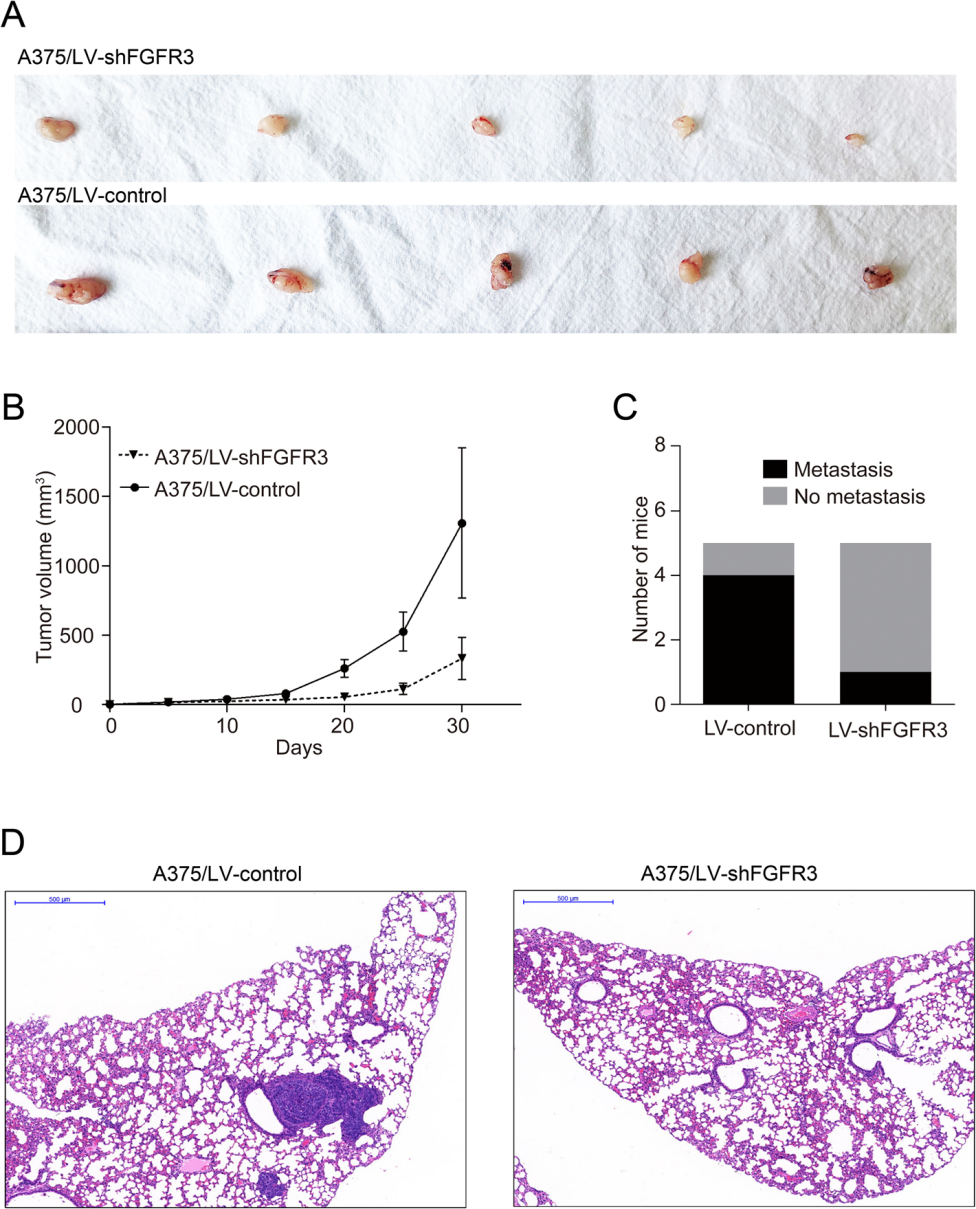
The growth and metastasis of A357 tumors in vivo. **a** tumors dissected from the mice with the xenograft A357 cells transfected with LV-shFGFR3 and A375/LV-control; **b** tumor growth curve derived from the xenograft A357 cells transfected with LV-shFGFR3 and A375/LV-control (** *p* < 0.05); **c** comparison of the numbers of animals with metastatic lung nodules between the animals injected with LV-shFGFR3 transfected A357 cells and the animals injected with LV-control transfected A357 cells; **d** lungs with metastatic lung nodules

A375/LV-shFGFR3 and A375/LV-control cells were injected into the right flank of nude mice and the tumor size was monitored for 5 weeks. The subcutaneous tumors induced by A375/LV-shFGFR3 cells were significantly (*p* < 0.05) smaller than those tumors induced by A375/LV-control cells (Fig. 5a-b). As for the lung metastasis assay, A375/LV-shFGFR3 cells and A375/LV-control cells were intravenously injected into nude mice. The development of metastatic lung nodules was monitored for 2 months. Knockdown of FGFR3 in A375 cells significantly (*p* < 0.05) reduced the number of mice with metastatic lung nodules (Fig. 5c-d).

### Knockdown of FGFR3 decreased the levels of N-cadherin, vimentin, p-ERK, p-AKT, and p-EGFR and increased the level of E-cadherin in vitro and in vivo

**Fig. 6:**
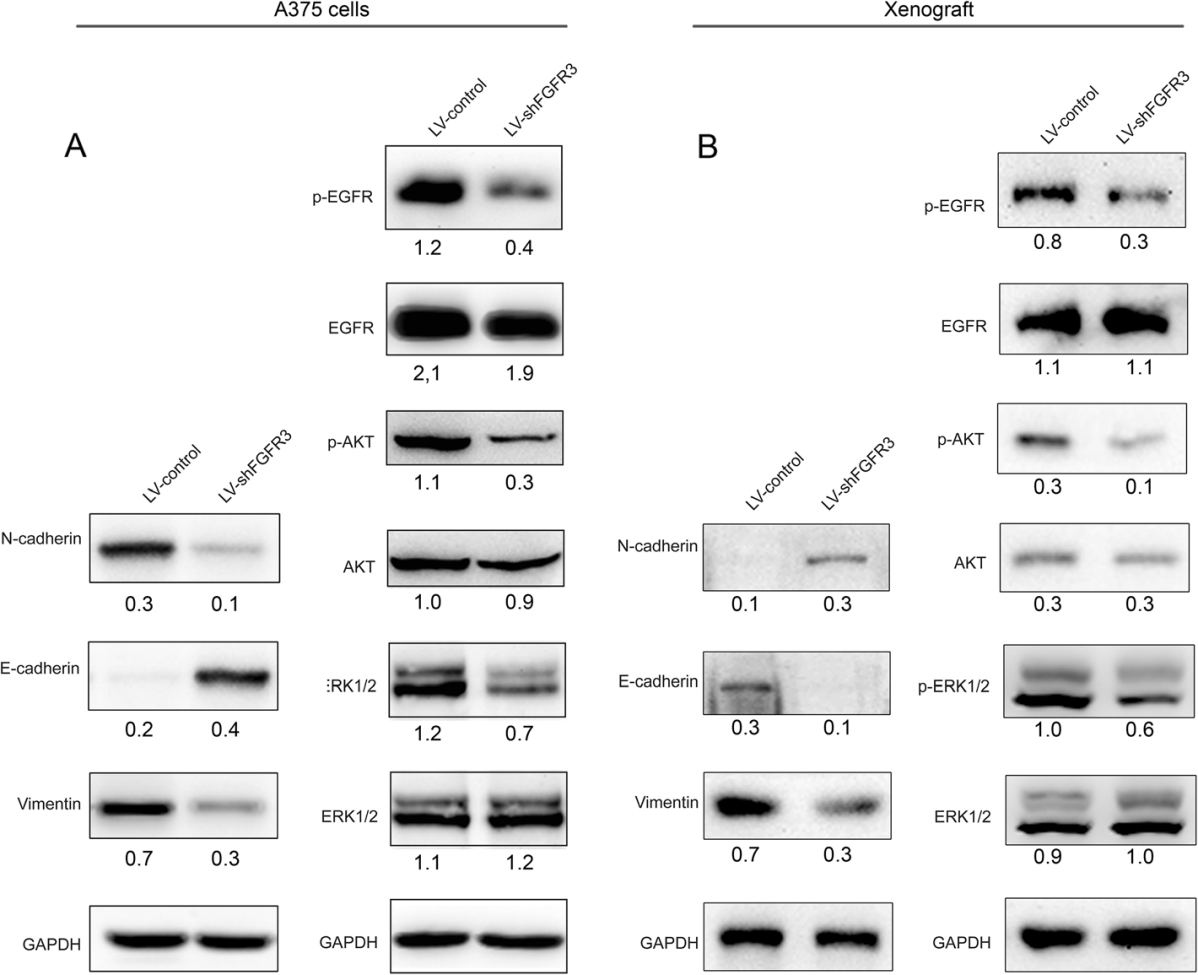
Protein expression of E-cadherin, N-cadherin, vimentin, ERK, AKT, and EGFR in the FGFR3 knockdown A357 cells and mice xenografts. **a** Western blot of E-cadherin, N-cadherin, vimentin, ERK, AKT, EGFR, p-ERK, p-AKT, and p-EGFR in A357 cells transfected with LV-shFGFR3 or LV-control; **b** Western blot of E-cadherin, N-cadherin, vimentin, ERK, AKT, EGFR, p-ERK, p-AKT, and p-EGFR in LV-shFGFR3 transfected A357 cell xenografts. The number under each band represents the relative density of the band normalized with the density of GAPDH

E-cadherin expression increased in the melanoma cells, while the expression of N-cadherin and vimentin decreased after the knockdown of FGFR3 in A357 cells (Fig. 6a). The expression of ERK, AKT, and EGFR showed minimal change after the knockdown of FGFR3 in A357 cells. However, the phosphorylation levels of these proteins dramatically decreased (Fig. 6a) after FGFR3 was knocked down. To validate the in vitro findings, we further analyzed the protein expression of genes mentioned above using a xenograft model of CMM by Western blot analysis. As the result, knockdown of FGFR3 suppressed the protein levels of p-ERK1/2, p-AKT, p-EGFR, vimentin and N-cadherin in vivo (Fig. 6b). Simultaneously, knockdown of FGFR3 also increased E-cadherin levels in vivo (Fig. 6b).

## Discussion

In this study, we found that FGFR3 was highly expressed in melanoma tissues. FGFR3 expression in CMM tissues was correlated with Breslow thickness and lymph node metastasis. FGFR3 promoted melanoma cell proliferation, colony formation, migration, and invasion in vitro. In addition, FGFR3 promoted the growth and metastasis of melanoma cells in vivo. The silencing of FGFR3 increased the expression of the epithelial marker E-cadherin, and reduced the levels of N-cadherin, vimentin, and phosphorylated ERK, AKT, and EGFR. These results demonstrate that FGFR3 promotes the growth and metastasis of melanoma through the EMT pathway and the phosphorylation of ERK, AKT, and EGFR.

Transfection of A357 cells with LV-shFGFR3 significantly decreased the mRNA and protein expression levels of FGFR3 by 75% in the cells. The transient transfection of A357 cells with pcDNA3.0-FGFR3 increased the mRNA and protein expression levels of FGFR3. These results indicate that FGFR3 expression was effectively altered by the gene intervention strategies used in this study.

In this study, FGFR3 expression was higher in melanoma tissues than the surrounding healthy tissues. FGFR3 expression in the CMM tissues was positively correlated with lymph node metastasis, which is in agreement with the observation that FGFR3 is expressed more in metastatic melanoma cells than primary tumor cells [28]. A FGFR3 activation mutation was associated with increased metastasis in many types of cancer. These results suggest that FGFR3 may play a vital role in the migration and invasion of melanoma cells, which makes it a potential biomarker for evaluating the risk of metastasis in melanoma patients.

FGFR3 expression was correlated with the Breslow thickness of melanoma, suggesting that FGFR3 may promote the growth of melanoma. This is supported by the FGFR3 knockdown study, as decreased FGFR3 expression decreased the colony formation and cell proliferation in A357 cells. In addition, the knockdown of FGFR3 increased cell apoptosis and the activity of caspase-3. When FGFR3 was overexpressed, colony formation and cell proliferation increased, which was in combination with decreased apoptosis of caspase-3 activity in the melanoma cells in vitro. The FGFR3 knockdown triggered a reduction in the size of tumors in mice xenografts, which further confirmed that FGFR3 is involved in the growth of melanoma.

FGFR3 expression in malignant melanoma tissues was correlated with lymph node metastasis, which is in agreement with the observation that FGFR3 is expressed more in metastatic melanoma cells than primary tumor cells [28], and that the activation mutation of FGFR3 can be associated with metastasis in many types of cancer. The knockdown of FGFR3 also decreased the cell migration and invasion abilities of the melanoma cells. However, FGFR3 overexpression increased the cell migration and invasion abilities in vitro. In addition, knockdown of FGFR3 in A375 cells reduced the number of mice with metastatic lung nodules. Together, all these results suggest that FGFR3 promotes the metastasis of melanoma.

The FGFR3 knockdown did not alter the expression of ERK and AKT. However, the phosphorylation levels of these proteins dramatically decreased, suggesting that FGFR3 modulates ERK and AKT at the post-translation level. FGFR3 is one of the four members of the FGFR family, which are transmembrane receptor tyrosine kinases (RTK) consisting of three immunoglobulin-like domains and one tyrosine kinase domain [3]. When fibroblast growth factor (FGF) binds to an FGFR, the receptor dimerizes, resulting in the transphosphorylation of the tyrosine kinase domains and activation of downstream signaling pathways. Through the intracellular FGFR substrate 2 (FRS2) and phospholipase Cg (PLC-g), the RAS/MAPK and PI3K/AKT signaling, STAT-dependent signaling, and RAS/MEK/ERK signaling pathways are activated. These pathways activate target genes in nucleus responsible for cell proliferation and survival [4]. These findings indicate that FGFR3 promotes the growth of melanoma through the PI3K/AKT and RAS/MEK/ERK signaling pathways by increasing the phosphorylation of ERK and AKT.

In this study, we found that the knockdown of FGFR3 only affected the phosphorylated form of EGFR. Activated EGFR stimulates cell proliferation, angiogenesis, migration, survival, and adhesion by activating the STAT signaling pathway, the KRAS-BRAF-MEK-ERK pathway, the PI3K and phospholipase C gamma protein pathway, and the AKT kinase pathway [24, 25]. FGFR3 may also promote the growth of melanoma through the EGFR signaling pathway by stimulating the phosphorylation of EGFR. While there is cross-talk between the EGFR and FGFR3 signaling pathways [29], it is unclear how FGFR3 modulates the phosphorylation of EGFR.

Epithelial-mesenchymal transition (EMT) is an indicator of metastatic potential [30] and is associated with aggressive cancers as it leads to enhanced cell migration and metastasis. E-cadherin, which is expressed in epithelial cells, is a transmembrane cell adhesion protein. E-cadherin is a tumor suppressor that inhibits cell invasion, and the loss of E-cadherin induces EMT [31]. N-cadherin is a mesenchymal cell type marker replaced by E-cadherin during EMT [32]. The mesenchymal marker vimentin is an intermediate filament, which takes the place of the epithelial cytokeratin filament [33]. The switch of cadherin involves the downregulation of E-cadherin by epithelial repressors (e.g., Snail) and upregulation of N-cadherin by mesenchymal activators (e.g., β-catenin) [34]. Both FGFR3 and EGFR modulate PI3K/Akt and ERK signaling pathways [4, 24, 25], and the activation of these signaling pathways promotes EMT [26]. In this study, FGFR3 knockdown increased the protein level of the epithelial marker E-cadherin, decreased the protein levels of mesenchymal markers N-cadherin and vimentin, and increased the levels of phosphorylated ERK, AKT, and EGFR in A357 cells. The FGFR3 knockdown also increased the migration and invasion properties of A357 cells in vitro and in vivo. Together, these results indicate that FGFR3 facilitates the metastasis of melanoma through ERK, AKT, and EGFR-activated EMT pathways.

## Conclusion

FGFR3 is highly expressed in malignant melanoma tissues and correlates with Breslow thickness and lymph node metastasis. FGFR3 regulates malignant melanoma growth, metastasis, and EMT behaviors by influencing the phosphorylation levels of ERK, AKT, and EGFR. FGFR3 may be an excellent biomarker for evaluating the risk of metastasis in melanoma patients.

## Data Availability

The datasets used and/or analyzed during the current study are available from the corresponding author on reasonable request.

## Abbreviations

CMM: Cutaneous malignant melanoma
EMT: Epithelial-mesenchymal transition
FGFR: Fibroblast growth factor receptors
SCC: Squamous cell carcinomas
STAT: Signal transducer and activator of transcription
UV: Ultraviolet
